# Possibly scalable solar hydrogen generation with quasi-artificial leaf approach

**DOI:** 10.1038/s41598-017-06849-x

**Published:** 2017-07-26

**Authors:** Kshirodra Kumar Patra, Bela D. Bhuskute, Chinnakonda S. Gopinath

**Affiliations:** 1 Catalysis Division, National Chemical Laboratory, Dr. Homi Bhabha Road, Pune, 411 008 India; 2 Network of Institutes for Solar Energy (NISE), NCL Campus, Pune, 411 008 India

## Abstract

Any solar energy harvesting technology must provide a net positive energy balance, and artificial leaf concept provided a platform for solar water splitting (SWS) towards that. However, device stability, high photocurrent generation, and scalability are the major challenges. A wireless device based on quasi-artificial leaf concept (QuAL), comprising Au on porous TiO_2_ electrode sensitized by PbS and CdS quantum dots (QD), was demonstrated to show sustainable solar hydrogen (490 ± 25 µmol/h (corresponds to 12 ml H_2_ h^−1^) from ~2 mg of photoanode material coated over 1 cm^2^ area with aqueous hole (S^2−^/SO_3_^2−^) scavenger. A linear extrapolation of the above results could lead to hydrogen production of 6 L/h.g over an area of ~23 × 23 cm^2^. Under one sun conditions, 4.3 mA/cm^2^ photocurrent generation, 5.6% power conversion efficiency, and spontaneous H_2_ generation were observed at no applied potential (see S1). A direct coupling of all components within themselves enhances the light absorption in the entire visible and NIR region and charge utilization. Thin film approach, as in DSSC, combined with porous titania enables networking of all the components of the device, and efficiently converts solar to chemical energy in a sustainable manner.

## Introduction

Like Steve Jobs, it may be essential to know ‘how to connect the seemingly (un)related dots? that could lead to a possible solution to the complex scientific problems. SWS is one such “Holy Grail” problem, as described by Alan Bard *et al*.^1^, which needs to network such (un)related dots. The conversion of solar energy into chemical energy through solar hydrogen production by artificial photosynthesis^2, 3^ is a highly promising approach, but an equally complex problem^4^. In this context, semiconductor oxide materials are the inevitable components for photocatalytic hydrogen generation. The efficiency of hydrogen production depends on the extent of light absorption, charge separation, charge migration, charge utilization at redox sites, and integrating all these factors in an efficient way^5, 6^. The optimization of all processes in a single photocatalyst that works in solar light with high activity, scalability, and sustainability has not been reported yet^6, 7^. Also the choice of the workable photocatalyst is limited to a few wide band gap semiconductors, like TiO_2_, which absorb in the ultraviolet (UV) region of the solar spectrum, and limiting the overall efficiency. Hence it is crucial to integrate (or network) the various components of light harvesting in an efficient manner towards higher efficiency.

As predicted by Alivisatos on 1996^8^, quantum dots (QDs) with tunable band gaps are currently employed for light emitting applications. Though QD is considered as a potential candidate for the light harvesting applications, such as SWS, it is yet to be established with high efficiency. Sensitization of a wide band gap semiconductor by a narrow band gap QD has been developed as a potential method for hydrogen generation^9^ due to light absorption in a broad wavelength (visible and near infrared) range. QDs are mostly used as a sensitizer due to large light absorption cross section, and shape and size dependent optical properties. Another way to improve the light absorption capacity of wide band gap semiconductor is to use plasmonic metal nanostructure, such as nanogold^10^. Such plasmonic metal nanostructure has been used to improve the efficiency of dye-sensitized solar cell (DSSC) and SWS^11, 12^. The enhancement in efficiency is due to the localized surface plasmon resonance (LSPR) of the metal nanoparticles (NPs). The frequency of LSPR oscillation depends on the shape and size of the metal nanostructure and dielectric constant of the surrounding medium^13, 14^. The oscillating electric field enhance the photocurrent generation in photoanodes by transferring its energy to the surrounding molecules or lattice. It is well known that the energy transfer from metal to semiconductor occurs by plasmon induced resonance energy transfer (PIRET) process^13–15^. PIRET occurs through a non-radiative energy transfer process from the dipole of the plasmonic metal NP to the dipole of the excited semiconductor in a limited area; hence it is critical that semiconductors must be in the immediate vicinity of plasmonic metal NP. It would be an added advantage if energy transfer from plasmon state can be utilized.

To take advantage of the above PIRET enhancement mechanism, we prepared a composite, in which the AuNPs are in physical proximity with titania as well as chalcogenides QDs for better solar light harvesting. QDs that are placed spatially very close to the AuNPs enhance the localized electric field surrounding the AuNPs and hence an increase in the photocurrent and solar hydrogen was observed by generating more electron-hole pairs in QDs. For this purpose, AuTiO_2_ nanocomposite was employed, where the plasmonic AuNPs are electronically integrated to the porous TiO_2_ surface, and further sensitized by PbS and CdS QDs^15, 16^. By using this concept, a wireless photochemical cell or quasi-artificial leaf (QuAL) was prepared to generate hydrogen without applying any potential. The success of this approach possibly would help to design better light harvesting synthetic architectures to produce solar fuels.

## Results and Discussion

AuNPs (~5 nm) was electronically integrated with TiO_2_ by following deposition-precipitation method^17^. The crystallographic facets of TiO_2_ remain unchanged before and after gold deposition, which is evident from the XRD results (Fig. S2). Au loading was varied between 0.02 and 0.084 wt%. 0.052 wt% Au containing AuTiO_2_ gives the maximum H_2_ yield (1.05 mMol/h.g) with methanol as sacrificial agent (Fig. S3), and hence this particular composite was used for the photoanode fabrication. PbS QDs was deposited on AuTiO_2_ electrode followed by CdS QDs by successive ionic layer adsorption and reaction (SILAR) technique^16^. By its very nature, SILAR method allows the physical proximity of chalcogenide QDs around Au nanoparticles. ZnS layer deposited finally on CdS QDs for providing stability to the photoanode, and it protects from photocorrosion. Detailed procedure for AuTiO_2_, photoanode fabrication, solar hydrogen production and various photoelectrochemical (PEC) measurements is given in S4. Various control photoanodes were also fabricated and evaluated.

**Figure 1:**
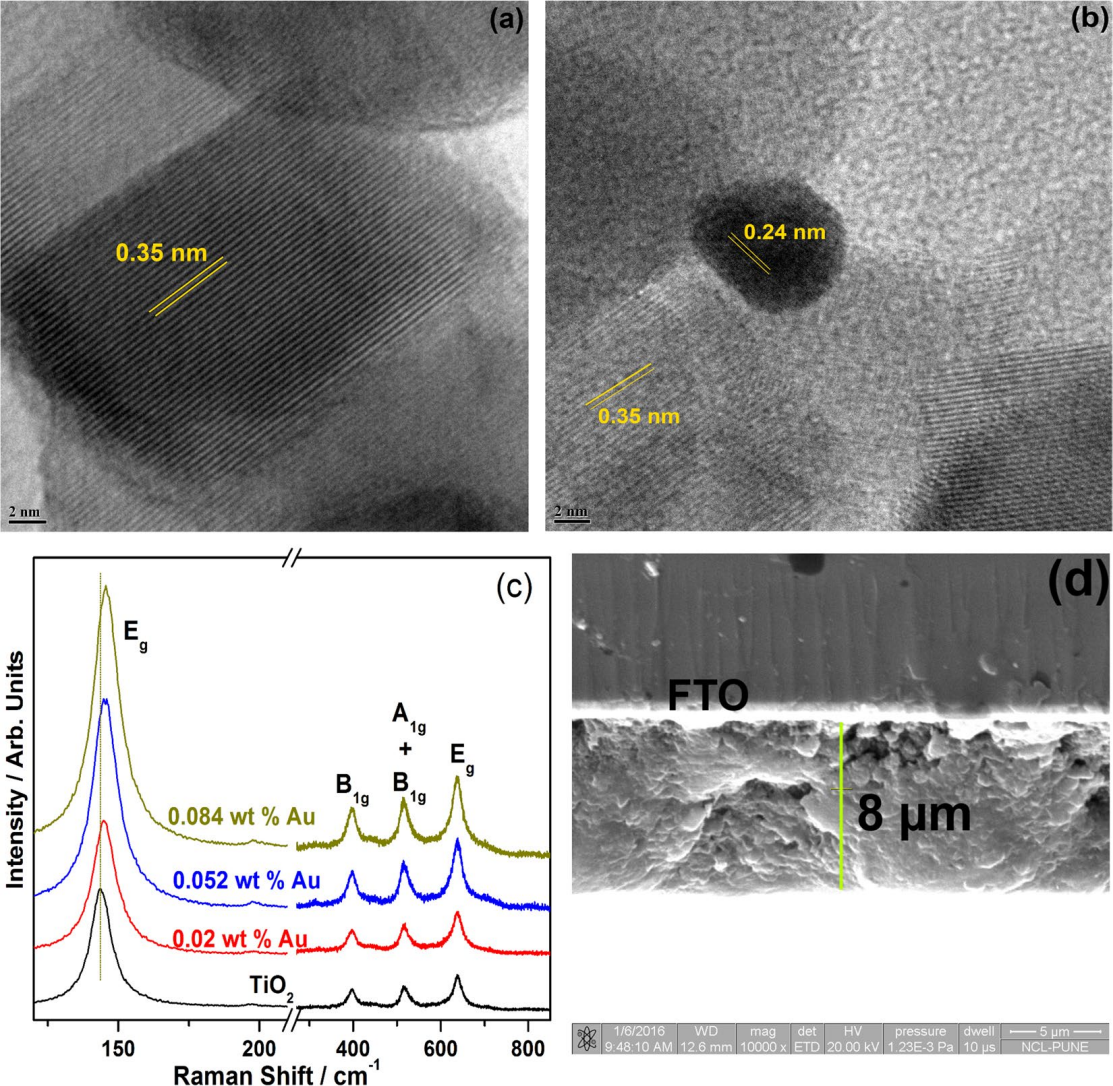
(**a** and **b**) HRTEM image of the AuTiO_2_ exhibiting the heterojunction between Au and TiO_2_. TiO_2_ particle exposes (101) facets predominantly. Scale bar in both images is 2 nm. (**c**) Raman spectra of pure TiO_2_ and Au-TiO_2_. Enhancement in the intensity of titania features and shift in E_g_ mode suggests the active role of PIRET process. (**d**) Representative image of freshly cleaved 8 µm thick surface of AuTiO_2_/PbS/CdS electrode recorded by SEM. The bright thick white line is due to FTO, coated on glass.

Electronic integration of AuNPs to the TiO_2_ surface is fully supported by Schottky junction from the representative HRTEM (Fig. 1a and b) and Raman spectroscopy (Fig. 1c) studies. HRTEM image shows the majority of lattice fringes corresponds to (101) crystallographic planes of anatase phase titania (d_101_ = 0.35 nm) (Fig. 1a). Figure 1b shows HRTEM image centered around a single gold nanoparticle surrounded by several titania particles. Uniform d = 0.24 nm value observed throughout the Au nanoparticle suggests the growth was along (111) facet. Au particle size varied in 6–8 nm range. As shown in Fig. 1b the metal-semiconductor heterojunction was observed with all composites, where the (111) facet of metal is in direct contact with (101) facets of several particles of anatase TiO_2_. It is known that heterojunction is an essential feature for separation of electron-hole pairs at the metal-semiconductor interface^5, 14^. Another advantage of Au-TiO_2_ heterojunction is the generation of additional charge carriers through PIRET process, which is expected to enhance photocurrent and hydrogen generation.

Raman active modes of anatase (145 (E_g_), 198 (E_g_), 398 (B_1g_), 516 (A_1g_ + B_1g_) and 640 cm^−1^ (E_g_)) and rutile (420–460 (E_g_) and 610 (A_1g_)) are observed in Fig. 1c; however, the frequency shift in the Raman spectra is attributed to the favorable binding interaction, particularly between (101) facet of anatase titania with the AuNPs. The electronic environment at the Au and TiO_2_ interface has been modified after Au integration, as a result of strong enhancement in intensity was observed for all anatase features in the Raman spectra. The increased intensity in Raman spectra after Au deposition is attributed to PIRET process resulting in a strong electronic interaction between Au and TiO_2_ which induces electron-hole pair separation in QDs efficiently.

The total thickness of the photoanode is ~8 µm, which is evident from the SEM analysis of *in situ* cleaved surface (Fig. 1d). It is to be particularly noted that no separate layers for deposited components were observed, rather a smooth layer above FTO layer was observed. This uniform layer is in contrast to the separate layers found for different components in the earlier reports^16^. We attribute this to the porous network of titania, which allows diffusion of Cd^2+^, S^2−^ and Pb^2+^ ions and hence the formation of CdS and PbS in the neighborhood of Au and TiO_2_ in Au-TiO_2_. Chemical mapping of Au, Cd, Ti, S, and Pb was measured on a freshly cleaved AuTiO_2_/PbS/CdS photoanode by FESEM-EDX, and the results are shown in Fig. S5. Throughout the cleaved photoanode film surface, all of the constituent elements can be seen; this fully supports the diffusion of Cd^2+^, S^2−^ and Pb^2+^ ions and chalcogenide formation occur in the pores of Au-TiO_2_. Relatively dense sulfur distribution from PbS and CdS ensures the Au is inevitably in their neighborhood. Uniform distribution fully asserts the physical proximity of Au and chalcogenide particles in the porous titania. Further, the physical proximity of various components in the confined pores suggests the formation of the abundant bulk heterostructure, which is expected to enhance solar light to current conversion efficiency.

Further support was obtained from the textural analysis of TiO_2_ and Au-TiO_2_, by low magnification TEM and porosity measurements by adsorption isotherms and the results are shown in Fig. S6. Mesoporous nature (type IV isotherm with H1 hysteresis) is clearly evident from the adsorption isotherm as well as TEM analysis. A marginal reduction in surface area and pore-size was observed from titania to AuTiO_2_ while maintaining the average pore size to be 8 nm.

**Figure 2:**
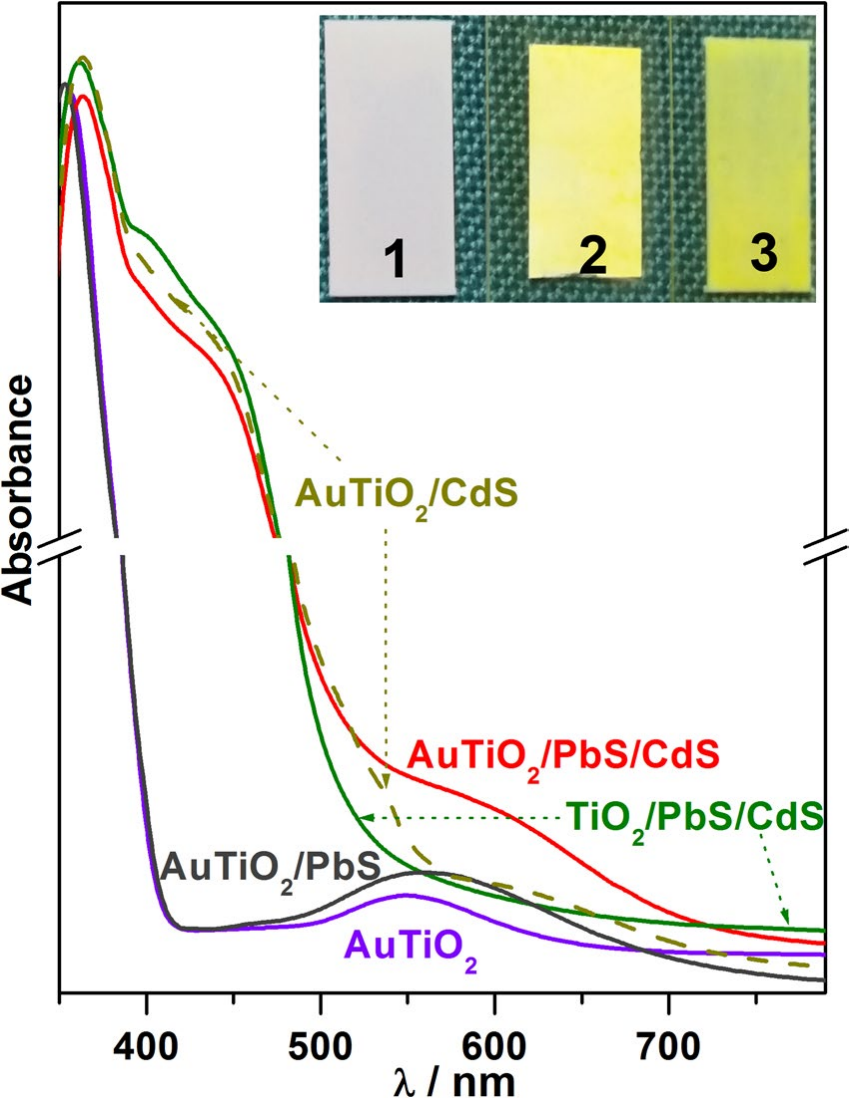
UV-Vis absorption spectra of the AuTiO_2_, AuTiO_2_/PbS, AuTiO_2_/CdS, TiO_2_/PbS/CdS, and AuTiO_2_/PbS/CdS photoanodes. Note the shift in absorption onset from AuTiO_2_ at 560 nm to about 700 nm for AuTiO_2_/PbS/CdS. The inset shows a digital photograph of the colors associated with photoanode films, (1) AuTiO_2_ and (2) TiO_2_/PbS/CdS, and (3) AuTiO_2_/PbS/CdS.

Figure 2 shows the UV-Visible absorption spectra of various photoanodes prepared with TiO_2_ and AuTiO_2_. Pure AuTiO_2_ shows an absorption band at 550 nm corresponding to the LSPR of the AuNPs. A broad absorption band centered at 450 nm corresponds to the CdS QD. LSPR peak of Au NPs was observed to be shifted as well as broadened to high λ regime in chalcogenide containing photoanode compared to AuTiO_2_; this is likely due to the change in the dielectric constant of the surrounding environment^15, 18^. In fact, light absorption up to ~700 nm was observed directly supports the electronic interaction of chalcogenide layers with nano-Au. AuTiO_2_/PbS and AuTiO_2_/CdS films prepared separately shows a systematic shift and broadening of Au-LSPR absorption to higher λ reiterates the influence of electronic interaction of PbS or CdS with Au in AuTiO_2_. TiO_2_ being a mesoporous substrate with a surface area of 59 m^2^/g, dispersion of chalcogenide QDs into titania is expected, which increases the interaction among them. Inset in Fig. 2 displays a digital photograph to show the changes in the colour of the photoanodes, from (1) purple for Au-TiO_2_, (2) yellow for TiO_2_/PbS/CdS to (3) greenish-yellow for AuTiO_2_/PbS/CdS.

**Figure 3:**
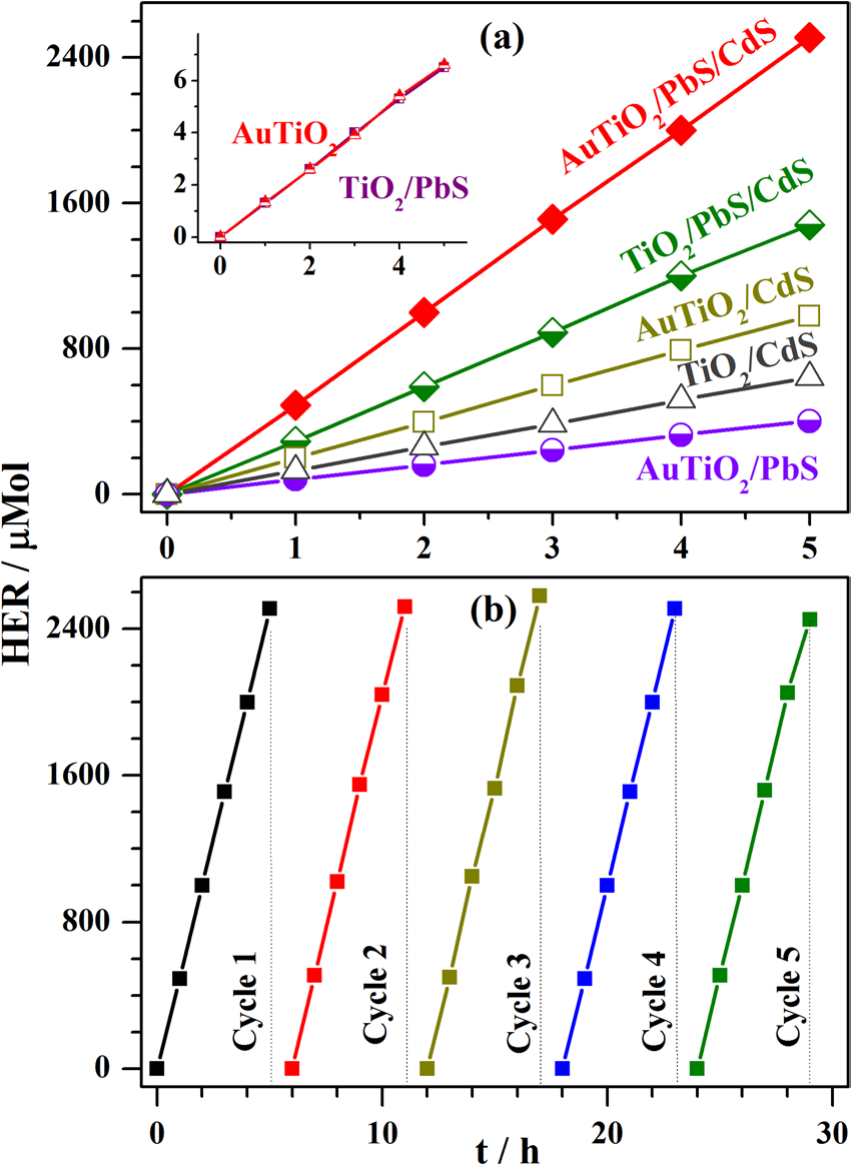
(**a**) Photocatalytic H_2_ evolution rate of wireless devices is shown under one sun illumination. Photochemical cell was immersed in the electrolyte (Na_2_S/Na_2_SO_3_) solution. (**b**) Photostability of the AuTiO_2_/PbS/CdS wireless device is demonstrated by performing the experiment for five cycles.

8 µm thick wireless photochemical cells were constructed with Au-TiO_2_ (Au-TiO_2_/PbS/CdS) or TiO_2_ (TiO_2_/PbS/CdS), and evaluated for solar hydrogen production. The total area of the photoactive material over FTO plate is 10 × 10 mm^2^. Pt was deposited as a strip (4 × 10 mm^2^) on the other half of the FTO plate, and it acts as a co-catalyst which provides the active sites for H_2_ evolution. The overall photochemical cell was immersed in the electrolyte (Na_2_S/Na_2_SO_3_) solution and illuminated under one sun condition (AM 1.5 filter, 100 mW/cm^2^) from the front side of the FTO plate. The weight of the photoanode material was carefully measured, and used to calculate the normalized photoactivity per gram with an assumption that the activity increased linearly. Figure 3a shows the H_2_ evolution rate (HER) by the wireless photochemical cells. The AuTiO_2_/PbS/CdS wireless photochemical cell exhibited highly enhanced H_2_ evolution rate (HER) at 490 ± 25 µmol/h and a PCE of 5.6%. Above HER value corresponds to 12 ± 0.5 ml/h H_2_ with 1 cm^2^ cell. A movie recorded under the measurement conditions (see S1) demonstrates the instant evolution of H_2_ bubbles predominantly from the Pt and Pt-photoactive material interface. Inverted gas burette was employed to collect H_2_ gas for quantification. Assuming a linear increase in HER with large area photoanodes, it is expected to provide about 6 L H_2_ for a gram of photoanode material coated over about 23 × 23 cm^2^. Even if there is a 50% (67%) decrease in HER at bigger size photoanodes (which is expected as in DSSC), it would still lead to 3 L (2 L) H_2_ h^−1^.g^−1^. It is to be underscored that the hydrogen yield expected after efficiency reduction considerations is still very significant and worth pursuing further. Nonetheless, higher area photoanodes needs to be systematically fabricated and evaluated, and we will address these issues in one of the future manuscripts. In the same manner solar hydrogen generation activity of TiO_2_/PbS/CdS was measured, and the result shows a significantly lower HER (0.3 mMol/h; 7.4 ± 0.4 ml/h from 1 cm^2^ cell). Similar work reported by Trevisan *et al*.^16^ shows only 0.18 ml/h H_2_ from 1 cm^2^ TiO_2_/PbS/CdS cell, indicating the efficacy of the present preparation method with optimized parameters (see S4).

Various control photoanodes prepared were also evaluated for HER, and the results are given in Fig. 3a. While TiO_2_/PbS and AuTiO_2_ shows a negligible HER (1.5 µmol/h; Fig. 2a inset), PbS on AuTiO_2_ demonstrating a quantum jump in HER (80 ± 5 µmol/h) underscores the role of PIRET process. Similarly, CdS on AuTiO_2_ also shows higher HER (200 ± 10 µmol/h) than on TiO_2_ (125 ± 10 µmol/h) again underscores the role of Au LSPR.

The stability of the wireless AuTiO_2_/PbS/CdS device was studied and the HER evaluated is shown in Fig. 3b. The device was continuously irradiated for 25 h, with about 1 h break after every five hours to replenish with the fresh sacrificial agent solution. Same activity was maintained in all cycles indicating that the photochemical cell is intact and devoid of any photocorrosion. Indeed, this is important given the presence of chalcogenide in the photochemical cell.

**Figure 4:**
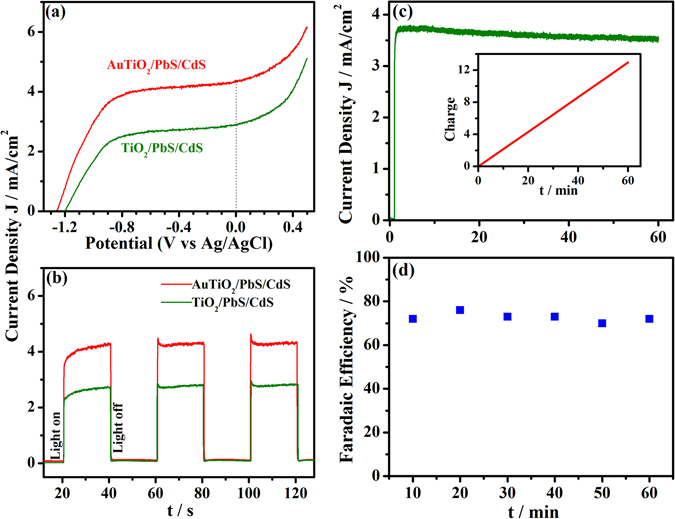
Photoelectrochemical performance of the AuTiO_2_/PbS/CdS and TiO_2_/PbS/CdS photoanode under one sun illumination. (**a**) j-V curves obtained under linear sweep voltammetry; (**b**) Chronoamperometry measurement at 0 V. (**c**) Chronoamperometric measurement shows the stability of photoanode for 1 h in the wired configuration. The inset demonstrates the amount of charge passed through the external circuit for 1 h. (**d**) Faradaic efficiency of the process calculated by comparing the amount of charge passed through the circuit and the amount of H_2_ gas evolved.

The photoelectrochemical (PEC) performance of the photoanodes was studied in a three-way electrode system with Ag/AgCl as the reference electrode and Pt as the counter electrode, and the results are shown in Fig. 4a and b. All PEC studies are measured in an aqueous Na_2_S/Na_2_SO_3_ solution. The AuTiO_2_/PbS/CdS working electrode exhibited 4.3 mA/cm^2^ photocurrent at zero applied voltage (vs Ag/AgCl) and one sun illumination (AM1.5 filter, 100 mW/cm^2^). The onset potential, derived from the J-V curves, was −1.19 V and −1.26 V for TiO_2_/PbS/CdS and AuTiO_2_/PbS/CdS (vs Ag/AgCl), respectively. 70 mV negative shift is expected to enhance the H_2_ evolution due to Fermi level (E_F_) equilibration^14, 19^ between AuNPs and semiconductors. E_F_ equilibration occurs since the QDs are distributed throughout the AuTiO_2_ matrix, and hence the Au-TiO_2_ is in direct contact with the liquid electrolyte. The negative shift in the onset potential of AuTiO_2_/PbS/CdS also indicates a better charge separation, and it improves the PEC performance.

An immediate photo response of the photoanode was studied by chronoamperometry at no applied voltage, and the results are shown in Fig. 4b. As compared to TiO_2_/PbS/CdS (2.7 mA/cm^2^), AuTiO_2_/PbS/CdS exhibits high photocurrent density of 4.3 mA/cm^2^. An increase in photocurrent generation with latter photoanode by 160% than that of the former highlights the efficient light harvesting by a combination of AuTiO_2_ as well as chalcogenide QDs. The increase in the current is attributed to the PIRET process, which in turn increase the generation of charge carriers. It is also to be noted that HER increases linearly with photocurrent generated in both photoanodes underscoring the charge utilization are to a similar extent.

The performance of the PEC cell was studied in both wired, and wireless configurations to produce H_2_, but at no applied potential under one sun illumination (Fig. 4c and d). In the wired configuration either of the photoanode was connected to the Pt as the counter electrode. Under illumination, photocurrent flows from the working electrode to Pt to produce H_2_. Figure 4c shows the chronoamperometry result measured for 1 hour. Solar hydrogen was measured, as in Fig. 3, but with the above two electrode configuration at no applied potential. Amount of H_2_ evolved was measured with GC periodically for every 10 min. Figure S7 displays the H_2_ evolution by the AuTiO_2_/PbS/CdS in the wired configuration, and a linear increase in H_2_ production represents the stable catalyst performance. However, under identical conditions, HER decreased dramatically to an order of magnitude with wired configuration (48 µmol/h) compared to the wireless configuration (490 µmol/h). In fact, the photoanode exhibited a photon to energy conversion (PEC) efficiency of 0.5% in the wired configuration. Resistance associated with the external circuit, and the Pt foil used (against nano-Pt coated on the wireless configurations) is the two important reasons for the decrease in H_2_ production in the wired configuration.

The total amount of charge passed through the external circuit, and the amount of H_2_ evolved determined the Faradaic efficiency. The Faradaic efficiency of photoanode with respect to time is plotted in Fig. 4d, and a steady efficiency of 72 ± 2% was observed. This result reiterates that HER occurs at an order of magnitude higher under no applied bias in the wireless configuration. No significant H_2_ generation was found with TiO_2_/PbS/CdS photoanode in the wired configuration.

**Figure 5:**
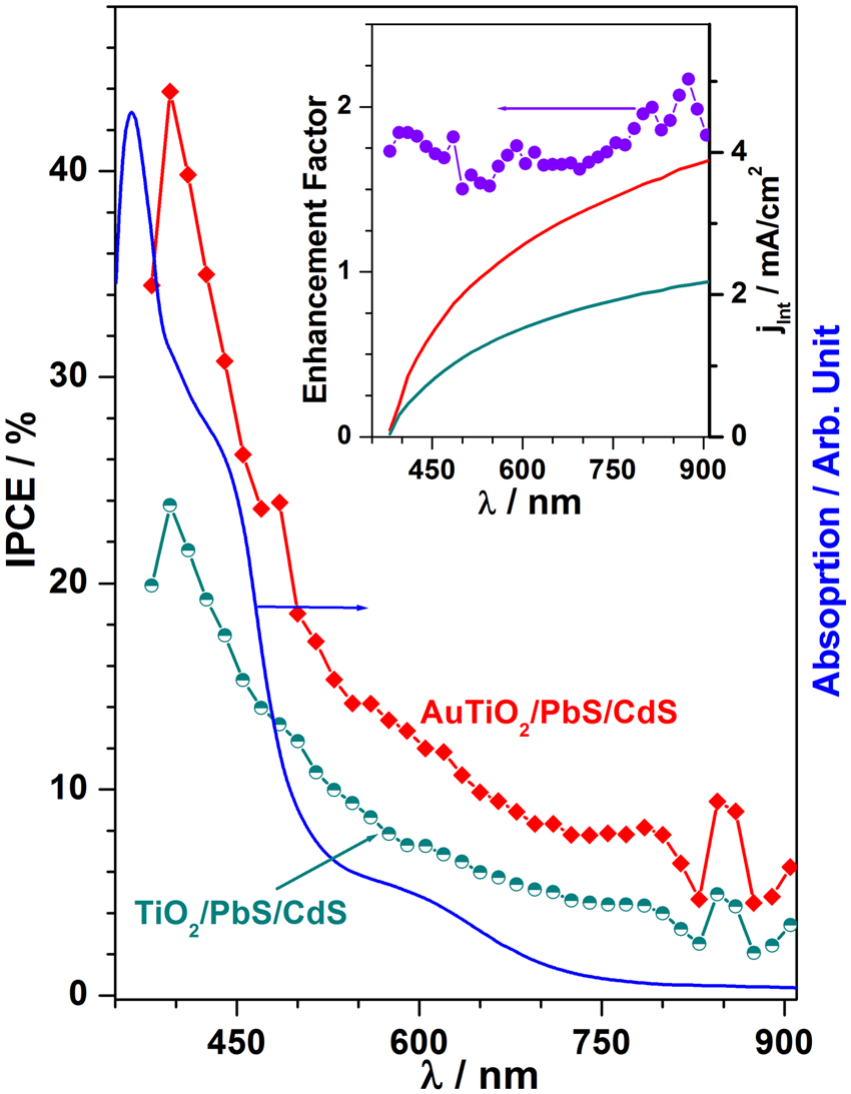
Wavelength dependent IPCE performance of the AuTiO_2_/PbS/CdS and TiO_2_/PbS/CdS photoanodes. Optical absorption of AuTiO_2_/PbS/CdS photoanode is also plotted for correlation between IPCE and light absorption. Inset shows the IPCE enhancement of AuTiO_2_/PbS/CdS over TiO_2_/PbS/CdS. An enhancement factor of 1.7–2 was observed over the entire absorption regime reiterates the active PIRET role in improving the current and HER. Integrated current obtained from IPCE for both photoanodes are also given.

PIRET effect is expected to increase the incident photoelectron conversion efficiency (IPCE) in the case of AuTiO_2_/PbS/CdS compared to TiO_2_/PbS/CdS. The IPCE spectrum was measured for both photoanodes at no applied voltage, and the result is shown in Fig. 5. An introduction of the Au nanoparticles improved the IPCE action spectrum markedly in the entire wavelength range from 400 to 900 nm. Optical absorption spectrum of AuTiO_2_/PbS/CdS (Fig. 5, blue trace) plotted fully supports the role of plasmon enhancement in IPCE. The IPCE at 450 nm was 27.4% and 15.2% for the photoanodes with and without Au nanoparticles, respectively. Critically, in the broadened nano gold plasmon absorption regime (500–700 nm), there is an improvement in IPCE with AuTiO_2_/PbS/CdS compared to TiO_2_/PbS/CdS. A good correspondence between IPCE and absorption spectrum is evident for AuTiO_2_/PbS/CdS and fully supports the role of PIRET in enhancing the IPCE between 500 and 700 nm. Significant contribution to IPCE (3–9%) from λ > 800 nm is also evident from the present results for both photoanodes, underscoring the near IR absorption. Integration of the IPCE spectrum over the entire wavelength region is shown in Fig. 5 (inset) leads to the total photocurrent of 3.9 and 2.2 mA/cm^2^ for AuTiO_2_/PbS/CdS and TiO_2_/PbS/CdS, respectively. This is in good agreement with the values (4.3 and 2.7 mA/cm^2^ for AuTiO_2_/PbS/CdS and TiO_2_/PbS/CdS, respectively) obtained in Fig. 4a. Inset in Fig. 5 also shows the IPCE enhancement factor, which was obtained by dividing the IPCE value of Au-containing photoanode to that of without Au at a given wavelength. An overall increase in enhancement factor was observed at all wavelengths up to 900 nm. Enhancement factor increases between 1.7 and 2. Notably an enhancement factor of 2 was observed for PbS and near IR absorption regimes further supporting the effective conversion of high λ light, which is good correspondence with that of ref. 16.

**Figure 6:**
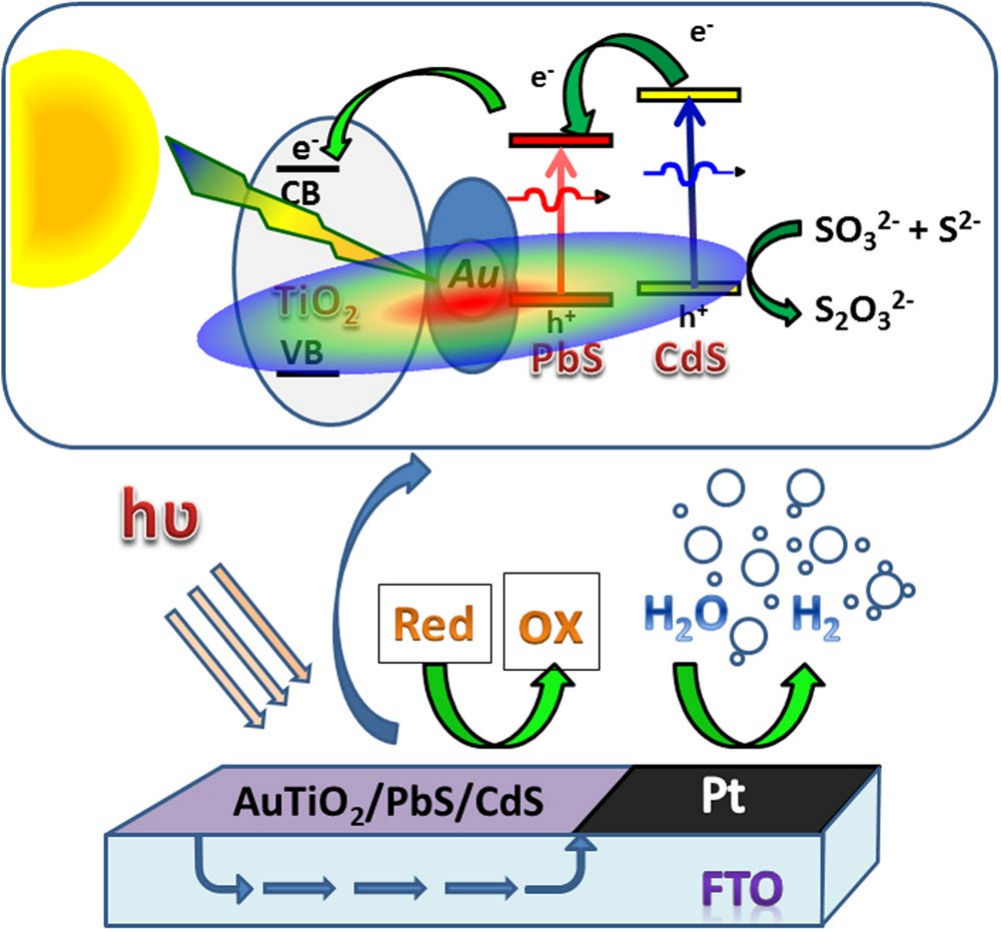
Schematic diagram for solar driven hydrogen evolution and the energy levels of different components. Au LSPR effect is represented by an ellipsoid.

Based on the H_2_ generation and characterization results obtained, a possible working mechanism of light harvesting is suggested in Fig. 6. The HRTEM and Raman spectral results (Fig. 1) reveal that the AuNPs is electronically integrated to the TiO_2_ surface and the PbS/CdS QDs are deposited on the TiO_2_ surface. Metal-semiconductor junction observed between Au and titania suggests the Schottky junctions, which helps in electron-hole pair separation. The shift in E_g_ band of titania to high wave number observed on Au deposition on TiO_2_ in Raman underscores the electronic integration among them. Visible light absorption from the entire visible light spectrum by Au-SPR and CdS, near-IR by PbS, at various wavelength ensures the maximum light absorption; without Au, this is restricted only to the corresponding wavelength regime. IPCE measurements are shown in Fig. 5 fully demonstrate the PIRET effect in the photoanode system with Au particles. The critical factor that increases the light absorption capacity of the photoactive material is due to the close spatial proximity of the QDs to the intensifying electric field surrounding the AuNPs in AuTiO_2_/PbS/CdS; this is shown in Fig. 6 with a VIBGYOR (Violet-Indigo-Blue-Green-Yellow-Orange-Red) ellipsoid extending on all semiconductors^20, 21^. The oscillating electric field thus generated enhance the photocurrent generation in photoanodes by transferring its energy to the surrounding chalcogenide and titania lattice. Due to the porous titania with gold particles anchored firmly on it, and the SILAR method employed for chalcogenide intercalation into those pores, gold is inevitably surrounded by one of the above components; it is evident from EDX chemical mapping given in Fig. S5. This factor ensures the gold nanoparticles efficiently transfer the energy through PIRET process and hence an overall increase in IPCE was observed throughout the visible and NIR wavelength range. Indeed this factor is entirely missing without Au. Porosity with TiO_2_ and SILAR method employed for PbS/CdS enhances the proximity of all components. As a result, the light absorption and electron-hole pair separation is also improved in the QDs, which leads to high catalytic activity with Au. The enhancement effect is attributed to the PIRET from the excited plasmonic NPs to the QDs.

## Conclusion

In conclusion, an attempt has been made to network the known factors that positively enhance light absorption to charge carrier utilization in the present communication. Current research efforts focus on using the AuNPs as a plasmonic sensitizer along with PbS/CdS QDs for designing wireless photochemical cell at no applied potential. It is also demonstrated that quasi-artificial leaf in wireless configuration harvests the solar light and converts it to H_2_ very efficiently than the wired configuration. Moist H_2_ produced from the quasi-artificial leaf can be directly fed to applications, like a fuel cell. Generally, the electronic integration of the plasmonic AuNPs and TiO_2_ with QDs sensitization provides a new pathway for better solar light harvesting. Further improvement in the more light absorption capacity of photoanode and earth-abundant co-catalyst would make the wireless photochemical cell more cost effective. Increasing the gold content, but without compromising the particle size, is expected to increase photocurrent generation and hydrogen in the present system. Replacement of Au by cheaper SPR metal, such as Ag, is a potential method to make it more economical. Fine tuning the porosity of titania could help further to improve the SWS efficiency by distributing the various light absorption components and its integration with titania. Simultaneous efforts are required to systematically scale up the photoanode size and evaluate them for longer period of time for moving towards real-world applications.

It is also essential to develop the counterpart of the present photoanode system to utilize the holes for oxygen generation. This would make the system complete towards overall water splitting and without any sacrificial agents. However, consistent efforts are required towards this direction.

## Methods

Several TiO_2_ or AuTiO_2_ electrodes (~8 μm thick) were fabricated first by well-known doctor-blade method^22^ to 1 cm^2^ area of 1 × 2 cm^2^ FTO plate. Above electrodes were kept at 40 °C for 12 h and subsequently calcined at 450 °C for 6 h. Rest of the electrode modification procedure remained the same and given for AuTiO_2_ electrode. The AuTiO_2_ thin film was sensitized with PbS QDs by SILAR method. 0.02 M aqueous solution of Pb(NO_3_)_2_ was used as a Pb^2+^ source and a 0.02 M Na_2_S.9H_2_O in methanol/ water (50/50 V/V) was used as a sulfide source. A single SILAR consists of immersion of the electrode into the lead precursor for 20 s and then rinsed with distilled water followed by immersion into sulfide precursor for 20 s. For CdS SILAR deposition, 0.05 M Cd(NO_3_)_2_ was used as Cd^2+^ source, and 0.02 M Na_2_S.9H2O in methanol/water (50/50 V/V) was used as a sulfide source. Two SILAR cycles for PbS followed by twelve SILAR cycles for CdS sensitization was applied on the titania electrodes. After PbS/CdS sensitization, the electrode was coated with three SILAR cycles of ZnS as the top-most layers. For this purpose, the AuTiO_2_/PbS/CdS electrode was dipped in 0.1 M aqueous solution of Zn(CH_3_COO)_2_ for 1 min., then rinsed with distilled water followed by dipping the electrode in Na_2_S solution for 1 min. ZnS is transparent to visible light, and it protects the device from photocorrosion. Various control photoanodes (such as AuTiO_2_/PbS, AuTiO_2_/CdS, AuTiO_2_, TiO_2_/PbS, and TiO_2_/CdS) were prepared by following the above method. Pt NPs was deposited, next to titania layer, by drop casting 5 mmol of chloroplatinic acid (H_2_PtCl_6_) (from Dyesol) over 0.4 × 1 cm^2^ area of FTO plate and evaluated for hydrogen generation and other studies. This results in the wireless device and employed for solar hydrogen production. AuTiO_2_ preparation method is given in detail in S4.

### Material characterization

The powder X-ray diffraction (XRD) patterns were acquired with PANalytical X’pert Pro dual goniometer diffractometer using Cu-Kα radiation (λ = 1.5406 Å) with a Ni-filter. Scanning electron microscopy (SEM) and energy dispersive X-ray (EDX) measurements were performed on a SEM system (Leica, Model Stereoscan-440) equipped with EDX analyzer (Bruker, D451-10C Quantax 200 with X-flash detector) attachment. HRTEM of the materials was conducted on a FEI TECNAI 3010 electron microscope operating at 300 kV (Cs = 0.6 mm; 1.6 Å resolution). Diffuse reflectance UV–Vis measurements were carried out on a Shimadzu spectrophotometer (model UV-2550) with spectral-grade BaSO_4_ as reference material. Raman spectra were recorded on a Horiba JY LabRAM HR 800 Raman spectrometer coupled with a microscope in reflectance mode with 633 nm excitation laser source, and a spectral resolution of 0.3 cm^−1^.

### Hydrogen evolution and photoelectrochemical measurements

In the wired configuration, the photoanode was dipped in a 250 ml three neck round bottom flask (RBF) containing 100 ml of 0.25 M Na_2_S and 0.35 M Na_2_SO_3_ (50/50 v/v) as sacrificial hole scavenger and Pt acts as a counter electrode, and the H_2_ evolution was studied at no applied potential. The evolved H_2_ was measured using GC (Agilent 7890A). The electrolyte was purged with N_2_ for 30 min. before every electrochemical experiment to remove the dissolved oxygen. For the wireless configuration, 8 ml of the electrolyte (0.25 M Na_2_S and 0.35 M Na_2_SO_3_) was used in a 50 ml RBF, and the photochemical cell was just dipped into the electrolyte with the front side being exposed to the light (see video given in S1 in supplementary information).

All the photoelectrochemical (PEC) measurements were performed in a three-way electrode system with Pt as a counter electrode and Ag/AgCl as the reference electrode. The chronoamperometry and LSV data were obtained by using a potentiostat (Gamry Reference 3000). A solar simulator coupled with AM 1.5 filter and 300 W Xe arc lamp (Newport instrument) was used as a light source for generating one sun condition for PEC and solar hydrogen generation experiments. The wavelength dependent IPCE measurements were performed with Newport solar simulator (UUX 1404565). Details about efficiency calculation are given in S4 in the supplementary information.

## Electronic supplementary material

Video for H2 generation

Supplementary Information
