# Supplementary material for: Possibly scalable solar hydrogen generation with quasi-artificial leaf approach
Source: Sci Rep. 2017 Jul 26;7:6515. doi: 10.1038/s41598-017-06849-x (PMC5529526; doi:10.1038/s41598-017-06849-x)
Supplement: Supplementary file 2 — Supplementary Information [file 41598_2017_6849_MOESM2_ESM.pdf]

Supplementary Information

Possibly scalable solar hydrogen generation with quasi-artificial leaf approach

Kshirodra Kumar Patra,[1] Bela D. Bhuskute,[1] and Chinnakonda S. Gopinath[1,2,*]

[1]Catalysis Division, National Chemical Laboratory, Dr. Homi Bhabha Road, Pune 411 008, India.

[2]Network of Institutes for Solar Energy (NISE), NCL Campus, Pune 411 008, India.

SI-1: Movie/video for Hydrogen generation under one sun conditions with no applied bias (or wireless configuration). VID_20160812_180514-SciRep.avi

SI-2: XRD: Powder XRD patterns in Figure S2 shows, the TiO_2 and Au/TiO_2samples with different weight percent of gold. No Au peak was observed in XRD patterns of Au/TiO_2 due to small amount gold dispersed on the TiO_2 surface.

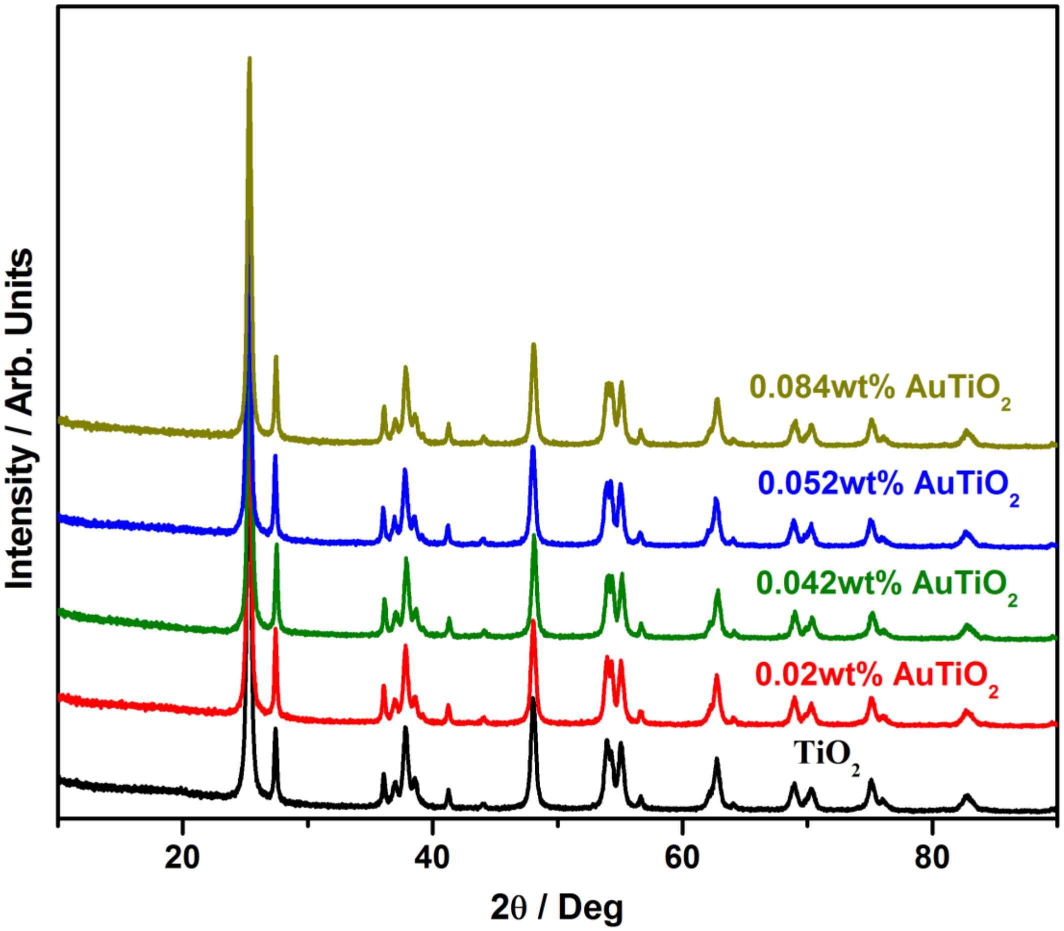

Figure S2. XRD patterns of TiO_2 and $AuTiO_2$.

SI-3: SWS activity with $AuTiO_2$:

SWS activity was measured with $AuTiO_2$ power with different Au-content (Fig. S3). The SWS was carried out by using 20 mg catalyst, 30 ml water and 10 ml methanol in a 50 ml quartz RB under one sun illumination condition. The maximum activity of 1.05 mMol/g/h of hydrogen was observed with 0.052 wt % of Au and the same wt % were used for the device fabrication.

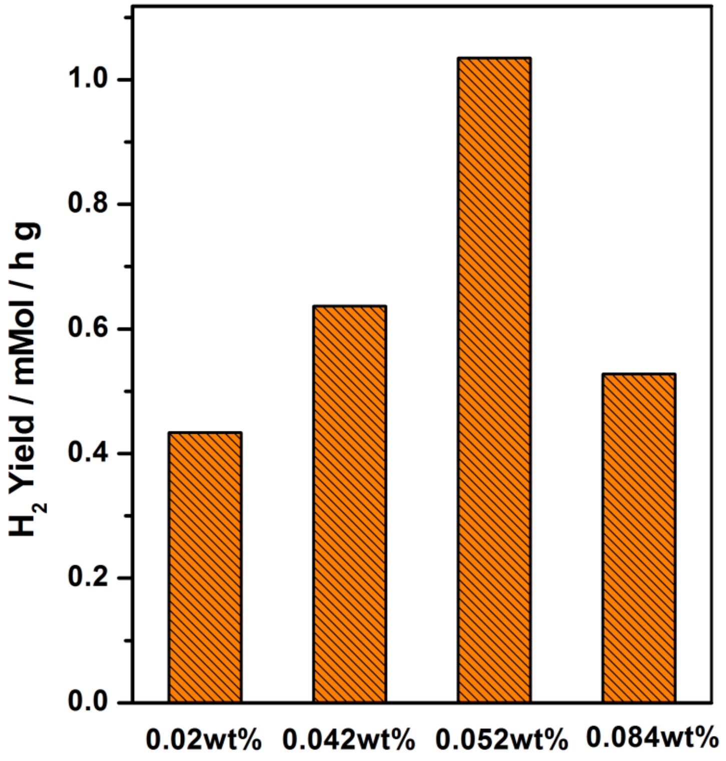

Figure S3.Photocatalytic activity of the $AuTiO_2$ with different Au loading in aqueous methanol solution under 1 sun condition.

SI-4: Experimental methods in detail

Fabrication of $AuTiO_2$/PbS/CdS photoanode. Different composition of $AuTiO_2$ was prepared by deposition precipitation method using urea as a basification agent. 500 mg TiO_2 (particle size 20 nm) was taken in to 250 ml beaker followed by the addition of 200 ml distilled water. Then 600 mg urea was added to the suspension under continuous stirring. Finally different weight of $HAuCl_4$ was added and kept at 80$^{\cdot 0}$C for 12 h under stirring condition. Then the solution was centrifuged, washed with ethanol and dried at 60$^{\cdot 0}$C. After drying the material was calcined in muffle furnace at 450$^{\cdot}$ ^{0}C for 2 h at a ramping rate of 5$^{\cdot}$C min^{-1}. Then the calcined material was ground and used for any characterization. The paste, for fabricating photochemical cell was

prepared by taking 3 g $AuTiO_2$ powder and 65 ml ethanol (large quantity of materials allows to make several photoanodes in one attempt with same/similar quality; however, smaller quantity can also be used to make fewer thin films) was added followed by 30 min stirring and 30 min sonication. Then 10 g terpenol was added and again stirred for 30 min. Finally 1.5 g of ethyl cellulose was added and stirred for 30 min. Then the ethanol was removed by a rotary evaporator at $60^{\cdot 0}C$. A doctor-blade technique was utilized to produce photoanode films. Briefly, transparent conducting glass (SnO_2:F, FTO glass, 15 Ω^{-1}) was cleaned by sonication in ethanol, acetone and de-ionized water for 20 min. Then 8 μm thick $AuTiO_2$ electrodes were doctor-bladed onto FTO surfaces. The pastes were then kept at $40^{\cdot 0}C$ for 12 h before heating at $450^{\cdot 0}C$ for 60 min to remove polymers.

The $AuTiO_2$ thin film was sensitized with PbS QDs by SILAR technique. 0.02 M aqueous solution of $Pb(NO_3)_2$ was used as a Pb^{2+} source and a 0.02M $Na_2S.9H_2O$ in methanol/ water (50/50 V/V) was used as a sulfide source. A single SILAR consists of immersion of the electrode in to the lead precursor for 20 s and then rinsed with distilled water followed by immersion in to sulfide precursor for 20 s. For CdS SILAR deposition, 0.05M $Cd(NO_3)_2$ was used as Cd^{2+}source and 0.02M Na_2S.9H2O in methanol/water (50/50 V/V) was used as a sulfide source. Two SILAR and twelve SILAR cycles were applied for PbS and CdS sensitization, respectively. After PbS/CdS sensitization, the electrode was coated with three SILAR cycles of ZnS. For this purpose, the $AuTiO_2$/PbS/CdS electrode was dipped in 0.1M aqueous solution of $Zn(CH_3COO)_2$ for 1min.,then rinsed with distilled water followed by dipping the electrode in Na_2S solution for 1 min. ZnS is transparent to visible light and it protects the device from photocorrosion. Various control photoanodes (such as $AuTiO_2$/PbS, $AuTiO_2$/CdS, $AuTiO_2$, TiO_2/PbS, and TiO2/CdS) were prepared by following the above method and evaluated for SWS. It is also to be mentioned that cells prepared with different precursors for Pb^{2+}, Cd^{2+} and Zn^{2+} and different immersion time was evaluated for SWS, and the above procedure provides the optimum parameters. Chemical mapping measured on freshly cleaved photoanodes with FESEM-EDX instrument (FEI, NOVA Nano SEM 450)[1] and it demonstrates a uniform distribution of all the constituent elements.

Fabrication of the wireless photochemical cell. The $AuTiO_2$/PbS/CdS was fabricated for $1cm^2$ area over the conducting surface of the FTO. Pt NPs was deposited by drop casting 5 mmol of chloroplatinic acid (H_2PtCl_6) (from Dyesol) over 0.4x1 cm^2 area of FTO and calcined at

450$^{\cdot 0}$C for 15 min. However, hydrogen evolution occurs exclusively from the Pt at the chalcogenide-Pt interface.

H_2 evolution in the wired and wireless configuration. In the wired configuration the photoanode was dipped in a 250 ml three neck RB containing 100 ml of 0.25M Na_2S and 0.35M Na_2SO_3 (50/50 v/v) as sacrificial hole scavenger and Pt and acts as a counter electrodes, and the H_2 evolution was studied at 0V. The evolved H_2 was measured using GC (Agilent 7890A). The electrolyte was purged with N_2 for 30 min. before every electrochemical experiment to remove the dissolved oxygen. For the wireless configuration, 8 ml of the electrolyte (0.25M Na_2S and 0.35M Na_2SO_3 (50/50 v/v) as sacrificial hole scavenger) was used in a 50 ml RB and the photochemical cell was just dipped in to the electrolyte with the front side being exposed to the light.

Electrochemical measurement. All the photoelectrochemical measurements were performed in a three way electrode system with Pt as counter electrode and Ag/AgCl as the reference electrode. The chronoamperometry and LSV data was obtained by using a potentiostat (Gamry Reference 3000). A solar simulator coupled with AM 1.5 filter and 300W Xe arc lamp (Newport instrument) was used as a light source for generating one sun condition. The wavelength dependent IPCE measurements were performed with Newport solar simulator (UUX 1404565) equipped with 300 W Xe lamp. Further details are available in ref. 2.

Calculation of PEC and Faradaic efficiency.

The PEC of the solar driven hydrogen generation efficiency was calculated by the following equation

$$\eta = \frac{2 \times 0.21(V) \times N_{H2}(mol) \times 96485(C.mol^{-1})}{I(W.cm^{-2}) \times A(cm^{2}) \times t(sec)} \times 100\%$$

Where N_{H2} is the amount of evolved H_2 gas, A is the area of the electrode exposed to the light, I is the intensity of light and t is the time of reaction and 0.21V represents the thermodynamic reaction potential for oxidation of sacrificial reagent. The Faradaic efficiency was calculated by the following equation.[3,4]

$$\eta_{faradaic} = \frac{2 \times N_{H2}(mol) \times 96485\ (C.mol^{-1})}{Q(C)} \times 100\%$$

Where Q is the total amount of charge passed through the external circuit and N_{H2} is the amount of hydrogen produce in the same period of time.

SI-5 – FESEM-EDX chemical mapping of $AuTiO_2$/PbS/CdS photoanode

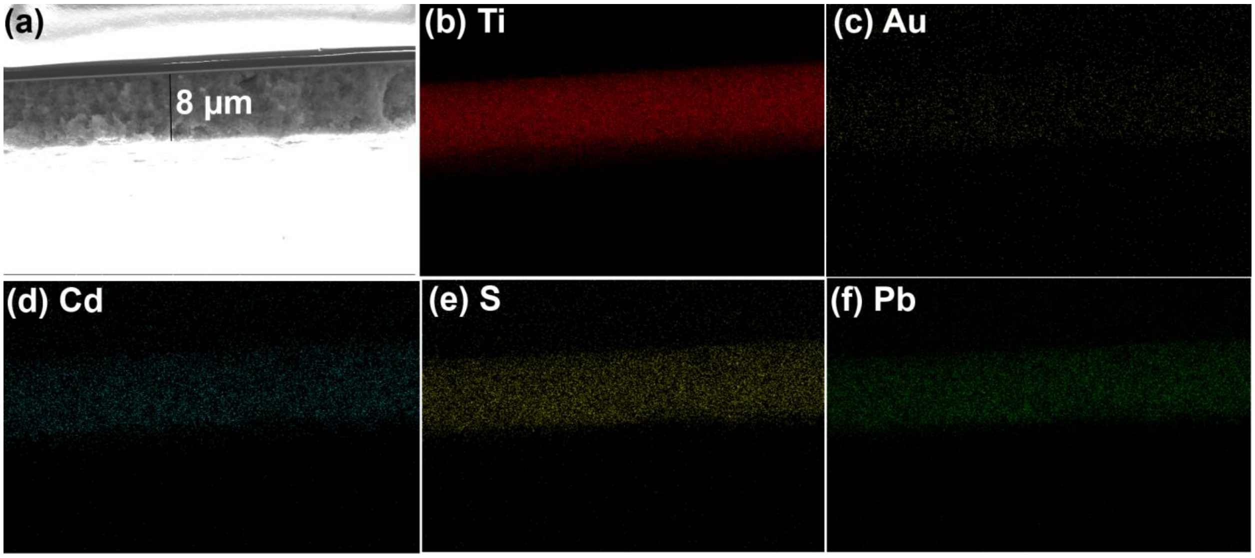

Figure S5: FESEM-EDX chemical mapping analysis carried out to show the uniform distribution of chalcogenides, and Au on TiO_2 in $AuTiO_2$/PbS/CdS photoanodes. Due to small amount of gold, faint yellow color can be seen throughout the film.

SI-6: Low resolution TEM, BET adsorption isotherm and pore-size distribution

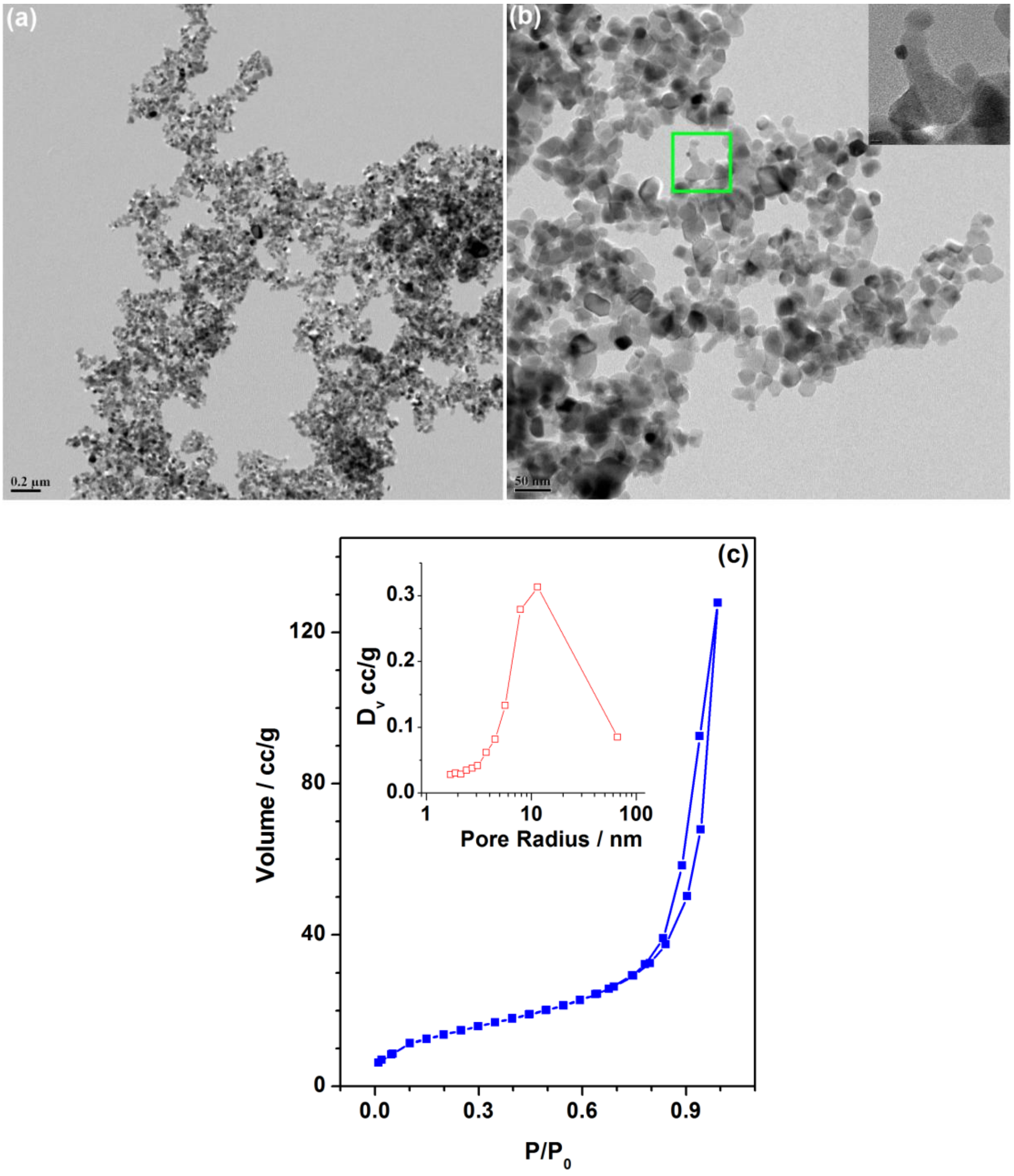

Figure S6: (a-b) Low resolution TEM images shown to support the porosity of the (a) titania, and (b) Au-TiO_2 employed in the present studies. Green square area (inset in b) was imaged separately to show the Au deposited on titania particle. (c) Adsorption-desorption isotherm (type IV with H1 hysteresis loop) and pore-size distribution measured at 77 K for titania fully demonstrates its mesoporous nature with majority of pore sizes between 4-12 nm. Au-TiO_2 also shows very similar pattern. Surface area for titania is 59 m^2/g, while that of Au-TiO_2 is 55 m^2/g. Average pore diameter was observed at 8 nm in both cases. However, a minor pore volume reduction occurs from 0.24 cc/g to 0.23 cc/g, without and with gold, respectively.

SI-7: Solar hydrogen in wired configuration.

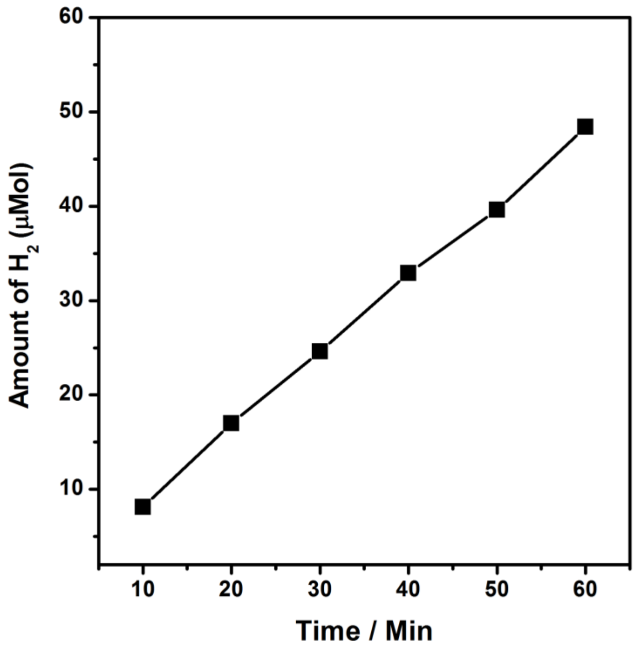

Figure S7. Solar driven hydrogen production with the AuTIO_2/PbS/CdS in the wired configuration at zero applied bias and the amount of H_2 gas evolved was measured by GC.

References

(1) Tathavadekar, M.C. et al., *Solar Energy* **112**, 12-19 (2015).

(2) Arulkashmir, A.; Sudhakar, V.; Krishnamoorthy, K., Band Edge Modulated Polymer Layer to Decrease Back Electron Transfer and Increase Efficiency in Sensitized Solar Cells, *Adv. Ener. Mater*. **6**, 1502334 (2016).

(3) Liu, C.; Tang, J.; Chen, H. M.; Liu, B.; Yang, P., A fully integrated nanosystem of semiconductor nanowires for direct solar water splitting. *Nano Lett.* **13**, 2989-2992 (2013).

(4) Raja, R. et al. Pt-free solar driven photoelectrochemical hydrogen fuel generation using 1T MoS_2 co-catalyst assembled CdS QDs/TiO_2 photoelectrode. *Chem. Commun.* **51**, 522-525 (2015).
